# Semaglutide Aggregates into Oligomeric Micelles and Short Fibrils in Aqueous Solution

**DOI:** 10.1021/acs.biomac.5c00342

**Published:** 2025-05-12

**Authors:** Ian W. Hamley, Lucas R. de Mello, Valeria Castelletto, Thomas Zinn, Nathan Cowieson, Jani Seitsonen, Thomas Bizien

**Affiliations:** † School of Chemistry, Food Biosciences and Pharmacy, 6816 University of Reading, Whiteknights, Reading, Berkshire RG6 6AD, U.K.; ‡ Diamond Light Source, 120796 Harwell Science and Innovation Campus, Didcot, Oxfordshire OX11 0DE, U.K.; § Nanomicroscopy Center, 174277 Aalto University, Puumiehenkuja 2, FIN-02150 Espoo, Finland; ∥ Synchrotron SOLEIL, L’Orme des Merisiers, Départementale 128, 91190 Saint-Aubin, France

## Abstract

Semaglutide is a lipopeptide with important applications in the treatment of diabetes, obesity, and other conditions. This class of drug (glucagon-like peptide-1 agonists and other lipidated peptides) may be susceptible to aggregation due to the tendency of lipopeptides to self-assemble into various nanostructures. Here, we show using cryogenic-TEM, small-angle X-ray scattering, and molecular dynamics simulations that semaglutide in aqueous solution undergoes slow aggregation into spherical micelles in water at sufficiently high concentration. A small population of needle-shaped fibril aggregates is also observed. At a lower concentration, dimer and trimer structures are formed. The micelles, once formed, are stable toward further aging. The aggregation influences the effect of semaglutide on the permeability of an epithelial gut model membrane of Caco-2 cells. These findings are expected to be important in understanding the long-term stability of semaglutide solutions and the potential effects of aggregation on therapeutic efficacy.

## Graphical abstract

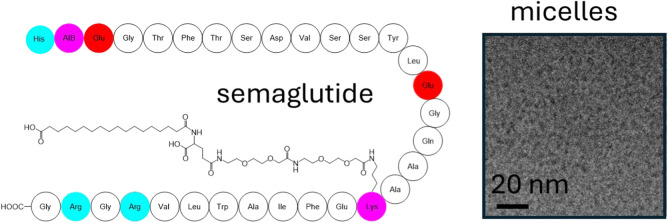

## Introduction

Gut hormones are biologically important peptides and include glucagon-like peptide-1 (GLP-1), which stimulates insulin production and suppresses glucagon secretion. Agonists of the GLP-1 receptor have recently emerged as powerful treatments not just for diabetes but also for obesity and potentially a range of other conditions such as cardiovascular disease. The GLP-1 analogues semaglutide and tirzepatide have recently gained immense attention for these applications.^1–6^ Semaglutide is marketed as Ozempic (injected solution) or Rybelsus (oral tablet form) for diabetes or Wegovy for weight loss, and tirzepatide is marketed as Mountjaro for diabetes treatment or Zepbound for weight loss. These molecules are derived from the native GLP-1 peptide sequence with substitution of several residues with non-natural amino acids to improve stability and reduce cleavage by specific enzymes. The structure of semaglutide, comprising 31 residues, is shown in Scheme 1. In addition, the molecule contains a lengthy carboxylic acid chain attached via an ethylene glycol-based spacer through the side chain of Lys20. The acylation (lipidation) facilitates serum albumin binding to increase circulation times in vivo.^1,2^ These structural modifications facilitated the original aim for semaglutide to develop an entity suitable for once-weekly administration.^1^

**Scheme 1:**
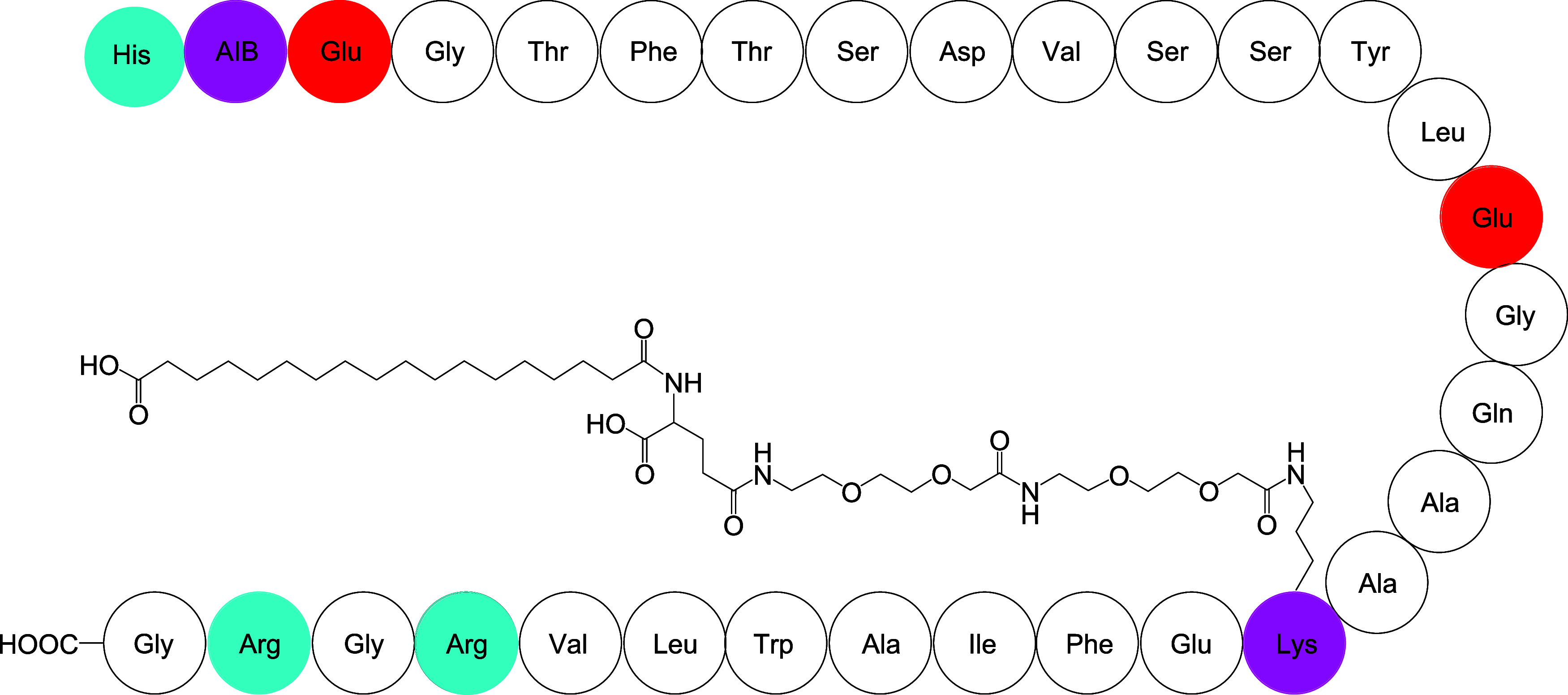
Molecular Structures of Semaglutide^a^

a Non-natural residues colored mauve (AIB = 2-aminoisobutyric acid), anionic red, and cationic cyan. A side chain attached to Lys20 molecules comprises a diEG_2_-γ-Glu linker (EG: ethylene glycol) and a C_18_ (octadecadienoic) carboxylic acid.

The attachment of lipid chains to peptide sequences in these lipopeptides (a type of peptide amphiphile) could be expected to impart an aggregation propensity due to the amphiphilicity of molecules containing hydrophobic chains and hydrophilic amino acids. The aggregation and self-assembly of many types of lipopeptides have been examined,^7,8^ and although there are now many studies on synthetic sequences (mainly with N-terminal lipidation), there are fewer studies on the self-assembly of lipidated gut hormone peptides.^9–13^ Human GLP-1 aggregates into ‘amyloid’ fibrils, the aggregation kinetics of which have been probed using Thioflavin T fluorescence.^9^ The nature of the off-pathway low-molecular-weight oligomers of GLP-1 and the C-terminal amide derivative has been examined in detail.^14^ It has been proposed that the lipidated GLP-1 derivative liraglutide (used to treat diabetics and obesity) forms small micelle-like oligomers, the association number being sensitive to pH near pH 7.^15^ Bothe et al. characterized pH-dependent oligomer states by size-exclusion chromatography and show a memory effect of initial oligomerization, depending on the pH at which lyophilization was performed.^16^ Subsequent fibrillization of liraglutide can be induced by mechanical stress (shaking).^16^ Later, Asymmetrical Flow Field-Flow Fractionation (AF4) studies suggest that the native state of liraglutide comprises pentameric oligomers.^17^ The effect of lipidation on the aggregation and stability of GLP-1 has been examined.^18^ Lipidation was found to reduce solubility (to a defined pH range) and lead to larger and more stable oligomers in comparison to the parent GLP-1 peptide. The lipidated analogues (including liraglutide-Am and semaglutide-Am) studied showed various aggregate structures, including extended fibrils and amorphous structures.^18^ The factors that affect the stability and aggregation of GLP-1 derivatives and other peptide therapeutics, including charge, sequence, pH, sequence, and concentration, have been discussed.^10^

There have been few studies to date on the aggregation of semaglutide. Venanzi et al. report on the basis of fluorescence probe measurements that a semaglutide derivative (not semaglutide itself, M. Venanzi, personal communication) forms aggregates above a critical aggregation concentration CAC = 20.2 μM (0.008 wt %) in phosphate buffer solution.^19^ The nature of the aggregates was probed via time-resolved tryptophan fluorescence and fluorescence anisotropy measurements as well as MD simulations, which indicate the initial formation of dimers followed by a slower process (over 40 days) of the formation of larger (mesoscopic) aggregates, characterized by a slight blue luminescence.^19^ Recently, Li et al. reported on the microemulsion formation (over a period of weeks) of semaglutide in phosphate buffer solution in the presence of hydrophobic surfaces.^20^ This leads to a characteristic ouzo appearance of the cloudy emulsified samples. The structure of the microemulsions was examined in detail by light scattering and SAXS, and the formation of microemulsion droplets was rationalized based on the Rayleigh model that balances surface charge and surface tension.

Here, we investigate the concentration dependence of the aggregation of semaglutide in water, comparing fresh and aged samples through a combination of cryogenic-TEM and SAXS. The conformational properties were probed using circular dichroism (CD) spectroscopy. SAXS and cryo-TEM reveal the formation of predominant well-defined spherical micelles after 40 days of aging in more concentrated samples (1 wt % aqueous solutions) with an additional small fraction of needle-shaped fibrils. In contrast, 0.1 wt % samples contain small oligomers (dimers and trimers) in aged solutions. CD spectra show the retention of the α-helical conformation of the peptide during the aging and micelle assembly process.

## Experimental Section

### Materials and Sample Preparation

Semaglutide was purchased from Bioserv (Calibre Scientific, Rotherham, UK). The purity by HPLC was >95%, and it was supplied in nonsalt (base) form. The molar mass is *M* = 4115.7 g mol^–1^ (4113.6 g mol^–1^ expected). Purity (by HPLC) is >98%. Characterization data are presented in Figures S1 and S2. Additional ESI-MS data showing the integrity of semaglutide after 40 days of aging are shown in Figure S3. No evidence for degradation in solution was observed.

Semaglutide powder was weighed, and the samples were prepared in glass vials using ultrapure water. To monitor the aggregation and stability, some samples were left to age up to 40 days in the fridge at 4 °C, protected from the light. The pH was observed just after preparation and after 40 days of incubation at 4 °C using pH strips due to the small volume of sample available. No evidence of microemulsion formation was observed. ^20^ pH values were recorded as follows: 1 wt % fresh pH 5.5, 1 wt % aged: pH 7, 0.1 wt % fresh: pH 6.5, 0.1 wt % aged: pH 7. It is notable that the pH crosses the p*K*_a_ of the N-terminal histidine (p*K*_a_ = 6 is the accepted value for isolated histidine) for the more concentrated solution upon aging.

For samples intended for use in culture media, sterile peptide powder was used to prepare highly concentrated samples in autoclaved ultrapure water and diluted in buffered culture media with the pH fixed at 7.4. The same process of aging at 4 °C for 40 days was used for stock sterile samples in water that were also dissolved in fresh culture media for assays intended to evaluate the permeability of living cells to semaglutide stored for longer periods of time.

#### Circular Dichroism (CD) Spectroscopy

Far-UV CD spectra were collected by using a Chirascan spectropolarimeter (Applied Photophysics, Leatherhead, UK) equipped with a thermal controller. Spectra were recorded from 180 to 400 nm. Samples were mounted in a quartz cell with detachable windows, with a path length of 0.01 mm. The CD spectra from the samples were corrected by water background subtraction. The CD spectra were smoothed using Chirascan Software for data analysis. The residue of the calculation was chosen to oscillate around the average to avoid artifacts in the smoothed curve. CD data, measured in mdeg, was normalized to molar ellipticity using the molar concentration of the sample and the cell path length.

##### Quantitative Analysis of α-Helix Content from CD Spectra

Semaglutide has 31 residues and a molar mass of 4113.6 g mol^–1^. The theoretical molar ellipticity at 222 nm (assuming 100% helicity) is then^13,21^

$${\left[\theta \right]}_{222}\left(\mathrm{theor}\right)=-37400\times \left(1-2.5/31\right)=-34384\;{\mathrm{deg cm}}^{2}\;{\mathrm{dmol}}^{-1}$$

The experimental values (and hence α-helix fractions *f*_a_) are as follows: For a fresh sample

$${\left[\theta \right]}_{222}\left(\mathrm{obs}\right)=-29310/31=-945.5\;{\mathrm{deg cm}}^{2}\;{\mathrm{dmol}}^{-1},,\mathrm{therefore}\;f_{a}=0.027$$

For a 40-day aged sample

$${\left[\theta \right]}_{222}\left(\mathrm{obs}\right)=-44286/31=-1428.6\;{\mathrm{deg cm}}^{2}{\mathrm{dmol}}^{-1},,\mathrm{therefore}{\;f}_{a}=0.042$$

#### Fluorescence Spectroscopy

Fluoroscence of tryptophan in semaglutide was excited at λ_exc_= 295 nm, and fluorescence emission spectra were measured using a Cary Eclipse spectrofluorometer (Agilent, Didcot, UK) with excitation and emission slits of 5 nm. The spectra were recorded from 300 to 600 nm. The temperature was maintained at 20 °C during the measurements. This protocol was performed using fresh Semaglutide samples and then repeated using the same samples stored at 4 °C for 40 days, protected from the light. To determine the critical aggregation concentration (CAC) of semaglutide using Nile red, Semaglutide samples (45-day aged) were prepared in 5 μM of Nile red. The wavelength for excitation was set at λ_exc_ = 550 nm with 5 nm windows, and spectra were collected from 570 to 750 nm.

#### Cryogenic-TEM (Cryo-TEM)

Imaging was carried out using a field emission cryo-electron microscope (JEOL JEM-3200FSC), operating at 200 kV. Images were taken in bright field mode and using zero loss energy filtering (omega type) with a slit width of 20 eV. Micrographs were recorded using a Gatan Ultrascan 4000 CCD camera. The specimen temperature was maintained at −187 °C during the imaging. Vitrified specimens were prepared by using an automated FEI Vitrobot device using Quantifoil 3.5/1 holey carbon copper grids with a hole size of 3.5 μm. Just prior to use, grids were plasma cleaned using a Gatan Solarus 9500 plasma cleaner and then transferred into the environmental chamber of a FEI Vitrobot at room temperature and 100% humidity. Thereafter, 3 μL of sample solution was applied on the grid, and it was blotted twice for 5 s and then vitrified in a 1/1 mixture of liquid ethane and propane at a temperature of −180 °C. The grids with vitrified sample solution were maintained at liquid nitrogen temperature and then cryo-transferred to the microscope.

#### Small-Angle X-ray Scattering (SAXS)

SAXS experiments were performed on beamline B21 ^22^ at Diamond Light Source (Harwell, UK) and SWING^23^ at synchrotron SOLEIL (Gif-sur-Yvette, France) and on the labSAXS instrument at Diamond Light Source.

On B21, the sample solutions were loaded into the 96-well plate of an EMBL BioSAXS robot and then injected via an automated sample exchanger into a quartz capillary (1.8 mm internal diameter) in the X-ray beam. The quartz capillary was enclosed in a vacuum chamber to avoid parasitic scattering. After the sample was injected into the capillary and reached the X-ray beam, the flow was stopped during the SAXS data acquisition. Beamline B21 operates with fixed camera length (3.9 m) and fixed energy (12.4 keV). The images were captured by using a PILATUS 2 M detector. Data processing was performed using the dedicated beamline software ScÅtter.

On SWING, the sample solutions were loaded into the 104-well plate of a custom-built BioSAXS robot^23,24^ and then injected via an automated sample exchanger into a quartz capillary (1.5 mm internal diameter) in the X-ray beam. The quartz capillary was enclosed in a vacuum chamber to avoid parasitic scattering. After the sample was injected into the capillary and reached the X-ray beam, the flow was stopped during the SAXS data acquisition. The beamline operates with a fixed camera length (3436 mm) and fixed energy (12.0 keV, i.e., wavelength λ = 1.033 Å). The images were captured by using an EIGER X4M detector. Data processing (masking, radial averaging, background subtraction) was performed by using dedicated beamline software FoxTrot. For each data set, 36 frames (0.99 s duration with 10 ms gap between frames) were acquired. Anomalous frames (resulting from insufficient sample injected in the beam, etc.) were not included in the background subtraction.

SAXS experiments (extending to higher *q*) were also performed using a lab-based Xeuss 3.0 (Xenocs, France) instrument at Diamond Light Source, equipped with a liquid gallium MetalJet X-ray source (Excillum, Sweden) with an energy of 9.24 keV, corresponding to a wavelength of 1.34 Å. SAXS patterns were measured for 28800 s with an incident X-ray photon flux of roughly 4.06 × 10^6^ ph/s on an EIGER2 R 1M detector (Dectris, Switzerland), and the sample-to-detector distance was set to 315 mm, giving a *q* range of 0.17–1.1 nm^–1^. The beam size is roughly 0.4 × 0.4 μm on the sample. The sample environment was a custom-designed low-noise flow-through cell having a thickness of roughly 0.7 mm and an area of 1.1 mm × 1.1 mm. The window material is silicon nitride. Silver behenate (layer spacing, *d* = 58.38 Å) was used to calibrate the SAXS data. SAXS images were analyzed using the IDL-based AXcess software package or software DAWN.

#### Molecular Dynamics Simulations

Molecular dynamics simulations were performed using Gromacs^25^ (versions 2023.2 and 2020.1-Ubuntu-2020.1-1). Semaglutide molecules were packed using Packmol^26^ into trimers (random placement of three molecules in a box) or spherical micelles (defined by positions of terminal atoms) with association numbers *p* = 15 or 30. The lipopeptide structures were generated by using UCSF Chimera. Simulations were performed using the CHARMM36 force field^27,28^ with manual patching of force field parameters for the Lys20 side chain based on parameters for the side chain treated as a “ligand” using CHARMM-GUI.^29,30^ The micelles were placed into simulation boxes (cubes) of length 14.2 nm, and systems were solvated using spc216 water. Each system was neutralized using a matching number of Na^+^ counterions. After energy minimization and 100 ps relaxation stages in the NVT and NPT ensembles, the final simulations were carried out in the NPT ensemble using a leapfrog integrator with steps of 1 fs up to 10000 ps (10 ns) in triplicate. The temperature was maintained at 300 K using the velocity-rescale (modified Berendsen) thermostat^31^ with a coupling constant of 10 steps. The pressure was maintained at 1 bar using the Parinello-Rahman barostat,^32^ and periodic boundary conditions were applied in all three dimensions. The Particle Mesh Ewald scheme^33,34^ was used for long-range electrostatics. Bonds were constrained using the LINCS algorithm,^35^ and the Verlet cutoff scheme^36^ was used. Coulomb and van der Waals cutoffs were 1.0 nm.

#### Transepithelial Electrical Resistance (TEER)

As a model of the gut epithelium, 1.5 × 10^5^ Caco-2 (ATCC, EUA) immortalized colorectal adenocarcinoma cells were seeded into 12 mm Millicell hanging cell culture transwells, with a PET lower membrane permeated by pores 0.4 μm in diameter. The transwells were then inserted into a 12-well plate to create a bicameral chamber system separated by the transwell PET membrane. The cells were cultivated until reaching confluence in DMEM supplemented with 10% FBS for at least 3 weeks before starting the TEER assay in order to form semipermeable membranes composed of Caco-2 cells separating the two chambers. For the TEER assay, a multimeter (TLC Electrical, UK). The microelectrodes were immobilized in a chopstick pattern and coated in carbon conductive ink to prevent corrosion, similar to previous reports using low-cost Volt-ohmeters for TEER.^37–40^

The resistance for the Caco-2 membrane tissue (*R*_tissue_) is measured in Ohms (Ω) and defined as^38^

$$R_{\mathrm{tissue}}=R_{\mathrm{Total}}-R_{\mathrm{Blank}}$$

This represents the total resistance of the system containing the semipermeable membranes minus the initial resistance from the cell containing only media or PBS. Since the resistance is inversely proportional to the area covered by the semipermeable membrane, the area of the transwell containing the membrane, *M*_Area_, has to be considered

$$\mathrm{TEER}=R_{\mathrm{tissue}}\left(\Omega \right)\times M_{\mathrm{Area}}\left({\mathrm{cm}}^{2}\right)$$

The area calculated for the transwells used in this assay is *M*_Area_ (cm^2^) = 1.12 cm^2^. Here, first, *R*_Blank_ was defined by measuring the resistance of control transwells without Caco-2 cells (DMEM only). The minimum TEER used as a cutoff for defining a Caco-2 cell membrane suitable for analysis varies in the literature, and we adopted a minimal value of TEER = 120 Ω·cm^2^ as a requirement for our assay, discarding transwells that presented lower values. The media used for this assay was DMEM without serum, and the temperature was fixed at 37 °C and pH fixed at 7.4 during TEER measurements. Aliquots of 0.02 wt % of semaglutide were added to wells in duplicate. The value of *R*_Total_ was measured using a chopstick electrode before adding semaglutide and every hour up to 6 h. After every measurement, the samples were returned to the CO_2_ incubator, and the last measurement was taken 26 h after the initial incubation with semaglutide. The TEER was calculated and is reported as % values relative to controls incubated without semaglutide.

## Results

Aggregation of semaglutide in aqueous solution was examined using a combination of cryogenic-TEM and small-angle X-ray scattering (SAXS), comparing fresh samples with aged samples, for the latter of which well-defined micellar structures (with a population of fibrils) were observed at a sufficiently high concentration, whereas smaller oligomers were observed at a lower concentration.

Cryo-TEM images for a freshly dissolved 1 wt % solution of semaglutide in water show an irregular clustered morphology (“fractal”-like) as shown in Figure 1a (additional images are shown in Figure S4). Cryo-TEM images for a 1 wt % semaglutide sample aged for 40 days are presented in Figure 1b (with additional cryo-TEM images shown in Figure S5). These images show the presence of very small micelle-like structures with a diameter of <5 nm, which coexist with isolated short needle-like fibrils. Potential further development in the morphology was probed by cryo-TEM for a sample aged for 79 days. We hypothesized that the fraction of fibril structures might increase as amyloid-like fibrillization is commonly observed for aggregating lipopeptides and peptides, including GLP-1 and derivatives.^9,18^ However, this was not observed, and indeed, cryo-TEM images indicate no further change in morphology compared to the 40-day aged sample; the structure still comprises micelle-like structures coexisting with a small fraction of needle-shaped fibrils (Figure S6).

**Figure 1:**
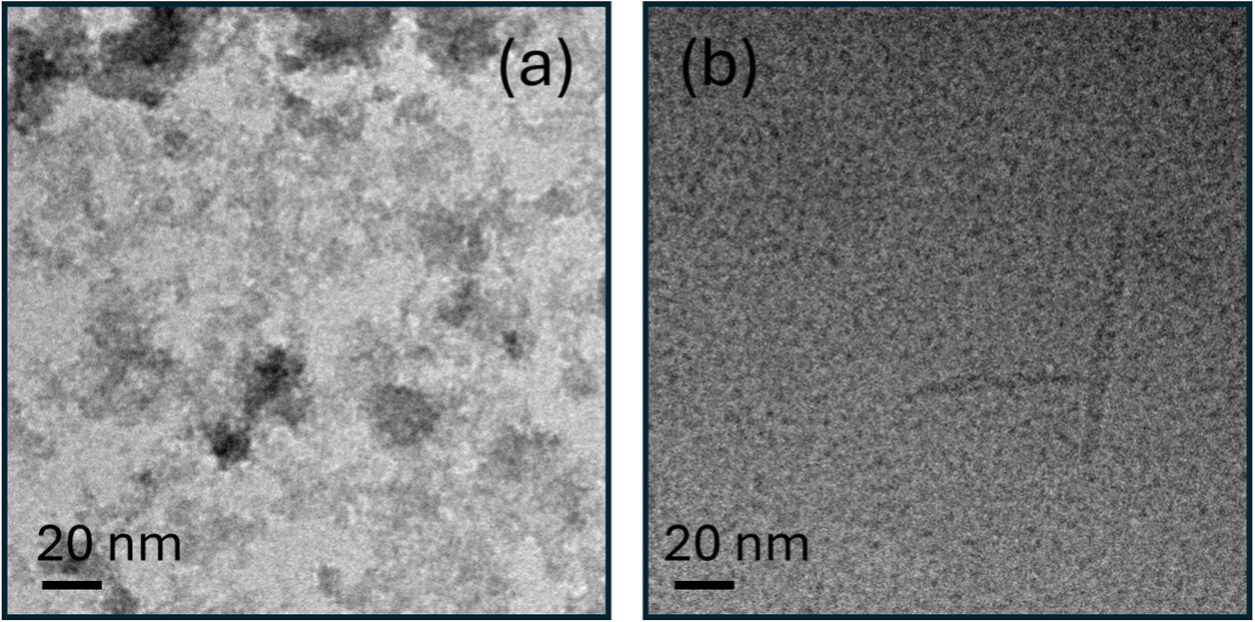
Cryo-TEM images for 1 wt % solutions of semaglutide: (a) freshly prepared solution and (b) solution aged for 40 days.

Cryo-TEM imaging was complemented with SAXS measurements, which provide quantitative information on the size, shape, and interactions between aggregate structures in solution. The SAXS data measured for several concentrations show changes in the intensity profiles upon aging, as illustrated in Figure 2a. The data at the lowest concentration (0.1 wt %) present relatively featureless scattering with a plateau extending to low *q*, from which the association number can be obtained (for aged samples) as detailed below. There is an increase in the scattering intensity for the aged sample. As evident from the data in Figure 2b, this data can be well fitted using a simple model for small oligomers/monomers, represented as “polymer coils”, i.e., Gaussian chains. The SAXS fit parameters are listed in Table S1. For fresh samples, an intensity decay at low wavenumber *q*, *I*(*q*) ∼ *q* ^–2.6^ is observed for lower concentration samples, when an extended *q* range is accessed (Figure S7). This scaling is characteristic of a mass fractal-like aggregate structure, consistent with the irregular aggregate structures in Figures 1a and S4. This scaling has been reported previously for semaglutide microemulsions, formed due to interactions with hydrophobic surfaces (so-called “ouzo” formation).^20^ Here, solutions were prepared in glass and were found to be transparent, and SAXS and cryo-TEM provide evidence for aggregation into irregular (not microemulsion-like) structures. It should be noted that the solution conditions differ in the previous work since samples were prepared in sodium phosphate buffer with NaCl, in contrast to the present studies in pure water.

**Figure 2:**
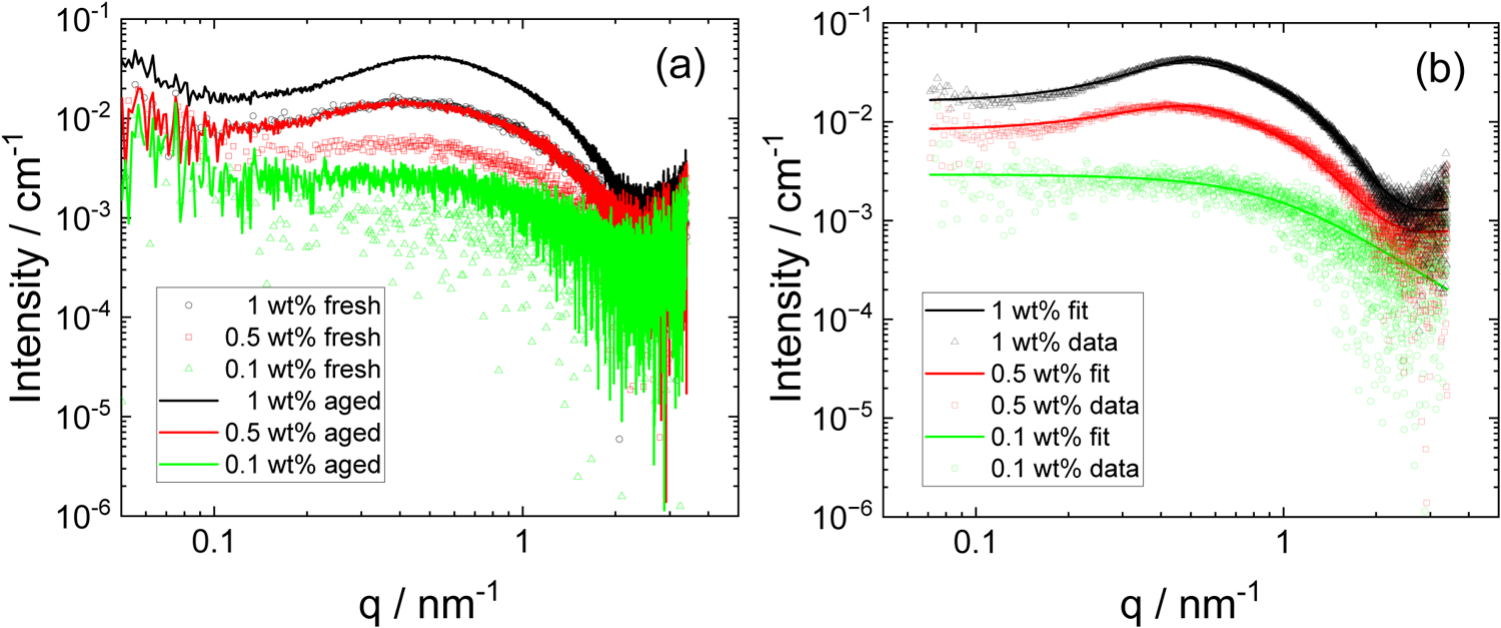
SAXS data. (a) Concentration dependence comparing fresh and aged samples (for clarity, only every third data point is shown for fresh samples), (b) SAXS data for 40-day aged samples (open symbols) with model form factor fits (solid lines) described in the text (fit parameters in Table S1).

A notable feature for the SAXS data for the higher concentration (0.5 and 1 wt %) samples is the enhancement upon aging of the structure factor peak centered at a wavenumber *q* = 0.4–0.5 nm^–1^. This is a signal of intermicellar correlations as the micelles revealed by cryo-TEM develop. This data is also shown in Figure 2b. This scattering can be well fitted using a combination of dominant structure factor term convoluted with a core–shell sphere form factor, chosen to represent the micelle structures revealed by cryo-TEM. To fit the structure factor peak, the Hayter–Penfold structure factor developed for charged colloids^41^ was used (as used by us to fit SAXS data from model lipopeptides with C_16_ lipid chains and short tripeptide sequences^42^). Attempts to fit this peak with the simpler hard sphere structure factor produced visibly poorer fits and unphysical fit parameters (very large volume fractions and too large hard sphere radii). The Hayter–Penfold model describes the data very well, and the fit parameters are listed in Table S1. The model provides the effective charge modulus per micelle, *z*_eff_ = 9.81 for a 0.5 wt % solution or *z*_eff_ = 10.8 for a 1 wt % solution. These are reasonable considering the estimated association number (discussed below) and noting that the molecules are expected to have a charge between −1 and 0, since semaglutide contains three cationic residues, two anionic residues, and two carboxyl groups, and the N-terminal histidine is close to its p*K*_a_ value at the measured pH of the aqueous solutions. The hard sphere radii from the structure factor are *R*_HS_ = 2.23–2.54 nm, which are close to the micelle outer radius from the form factor component of the SAXS fit (Table S1), and both values are consistent with the micelle size that can be estimated from cryo-TEM images. The radius of the inner core of the micelle from the form factor fits is *R*_i_ = 1.5 nm, which is reasonable considering the estimated length of the octadecanoyl chains (Scheme 1).

We performed additional SAXS measurements for a 1 wt % sample using a lab-based instrument extending to higher *q* than the synchrotron measurements, to probe form factor effects in more detail, and the data shown in Figure S8 show additional scattering at high *q* due to the micellar form factor.

The association number of the micelle can be obtained in a model-independent fashion from the forward scattering intensity of the SAXS data in dilute solution.^43,44^ The measured synchrotron SAXS data presented here is in absolute units (cm^–1^) and the forward scattering (at *q* = 0) can be written as^45^

1 $$I\left(0\right)=c_{\mathrm{mic}}M_{\mathrm{mic}}{\left[r_{0}v_{p}\left(\rho_{l}-\rho_{0}\right)\right]}^{2}/N_{A}$$

Here, *c*_mic_ is the concentration of micelles, *M*_mic_ is the micelle molar mass, *r*_0_ is the classical electron radius [0.28179 × 10^–12^ cm e ^–1^], *v*_p_ is the partial specific volume, and ρ_l_ and ρ_0_ represent the lipopeptide and solvent (water) electron density. Here, we wish to obtain the micelle molar mass and hence *p*. Rearranging eq 2 gives

2 $$M_{mic}=\frac{I\left(0\right)N_{A}}{c_{\mathrm{mic}}{\left[r_{0}v_{p}\left(\rho_{l}-\rho_{0}\right)\right]}^{2}}$$

We consider the data for 0.1 wt % semaglutide, where there is no evidence for structure factor effects. Using the equation due to Tanford for the volume per lipid chain,^46^ *v*_*l*_ = 27.4 + 26.9*n* (where *n* is the number of carbons in the lipid chain excluding the terminal CH_3_ group, i.e., *n* = 17 and *v*_*l*_ is in units of Å^3^) gives *v*_*l*_ = 487.4 Å^3^. Considering only methylene units, the tail contains 142 electrons in this volume, i.e., the electron density is ρ_l_= 0.291 e Å ^–3^ (which is close to the expected electron density for methylene groups in alkyl chains^47,48^ ). The electron density of water is taken as ρ_0_ = 0.333 e Å^–3^. For a concentration *c*_mic_ = 0.1 wt % (0.001 g cm^–3^), the forward scattering for aged samples is *I*(0) = 0.0025 cm^–1^ (with high reproducibility in measurements on different synchrotron beamlines, Figures 2a and S7). Substituting this into eq 2 with *v*_p_ = 293.5 cm^3^ mol^–1^/250 g mol^–1^ = 1.17 cm^3^ g^–1^ leads to *M*_mic_ = 9111 g mol^–1^, i.e., *p* = *M*_mic_/*M*_mol_ ≈ 2.2 [here, *M*_mol_ = 4113.6 g mol^–1^ is the molar mass of semaglutide]. Thus, in dilute solution after aging, SAXS reveals that semaglutide is present as small aggregates, specifically dimers and trimers (considering the estimated uncertainty on *p* given the approximations in the estimation of specific volume and lipid core volume and electron density). This is consistent with atomistic MD on a related semaglutide derivative.^19^ As a rough estimate, the ratio of the intensity at low *q*, *I*(*q* = 0.01) for 1 and 0.1 wt % is approximately 6; therefore, proportionally semaglutide micelles may comprise approximately *p* = 12–15 molecules. However, it should be noted that the presence of a significant structure factor peak in the data for 0.5 and 1 wt % solutions will adversely affect the reliability of this estimate. Molecular packing and molecular dynamics simulations, in fact, suggest that *p* = 30 is a more reasonable estimate. Representative configurations from atomistic MD simulation frames are shown in Figure 3. Figure 3a shows a trimer, in which the lipid side chains are partly exposed to the solvent; however, they are much more sequestered in the core of a *p* = 30 micelle shown in Figure 3b, which was found to be stable during the MD simulation. An image of a micelle with lower *p* = 15 from MD simulations is shown in Figure S9, and while stable during the MD run, the molecules are not well packed, and significant regions of solvent-exposed hydrophobic chains are observed, which is considered unphysical. Data for the solvent accessible surface area and other micelle properties for micelles shown in Figure 3b (*p* = 30) are shown in Figure S10. The aggregation propensity (AP) may be defined as the ratio of initial-to-final SASA,^49^ and here, AP = 1.36, which indicates good aggregation propensity. The simulation configurations were used to compute electron density profiles as shown in Figure 3c; the profile for the semaglutide micelles shows a limiting electron density in the micelle core ρ_l_ = 0.25–0.30 e Å ^–3^, which is close to the expected electron density for methylene groups in alkyl chains.^47,48^ This, together with visualization of the location of Lys20 in the micelle core (Figure 3b), shows that self-assembly is driven by the sequestering of the lipid chains in the micelle core, consistent with the behavior of other amphiphilic micelle-forming molecules such as various lipids. It is ascribed both to the aggregation of the lipid chains in the micelle core and to the partitioning of the hydrophilic residues in the micelle corona. Micellization is typically the result of the hydrophobic effect in which the entropy of packing of the water molecules is increased arising from the disruption of local hydrogen-bonding networks due to micelle formation.^50,51^

**Figure 3:**
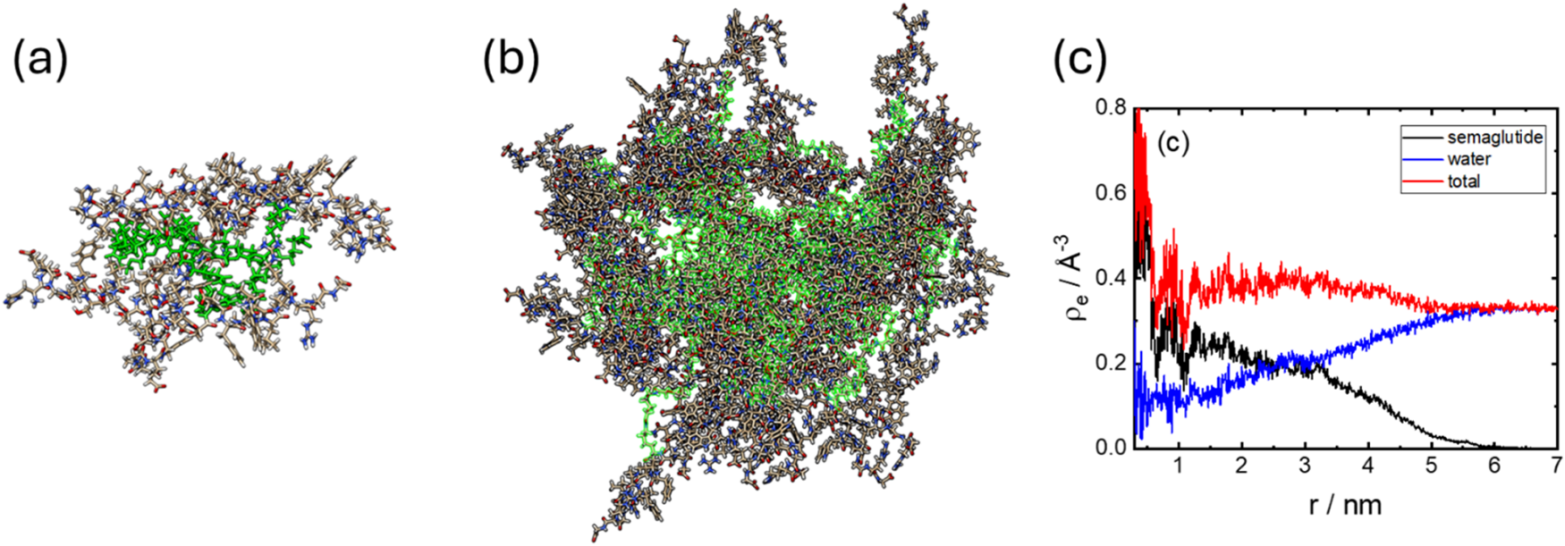
(a, b) Images of configurations after MD simulation for semaglutide aggregates, after aging. (a) Trimer formed at low concentration and (b) micelle with *p* = 30 formed at higher concentration. The green chains are highlighted lipidated side chains on Lys20. (c) Electron density profiles from MD simulations (average from last 100 ps).

The conformational properties of semaglutide were examined using circular dichroism (CD), investigating any aging effects. Spectra are presented in Figure S11, and it is clear that after aging, semaglutide (1 wt % solution) presents a classical α-helical CD spectrum,^52,53^ with a positive maximum at 190 nm and negative minima at 208 and 222 nm. The spectrum for the freshly dissolved sample does show the 208 and 222 nm minima but not the 190 nm maximum. The development of the α-helical conformation accompanies micellization, i.e., the outer part of the micelles comprises peptide in the α-helical conformation, while the inner part comprises the hydrophobic lipid chains (and hydrophilic diethylene glycol spacer). These findings differ from those reported for a semaglutide derivative, for which aged solutions show β-sheet pattern CD spectra.^19^ Since CD molar ellipticities for α-helices can be compared to those calculated for an ideal coil,^21^ we obtained fractional α-helix contents from [Θ]_222_, as detailed in the Experimental Section, which indicates an increase from 2.7% α-helix content to 4.2% upon aging. These low values arise from the disruption of the coil structure due to the lateral attachment of the lipidated chain at Lys20 (Scheme 1). Additionally, the ratio [Θ]_222_/[ Θ]_208_ provides an indication of coiled-coil formation,^13,21,54^ here [Θ]_222_/[ Θ]_208_ < 1, which points to the presence of isolated coils only. Since the sequence contains a tryptophan residue (Scheme 1), semaglutide and related derivatives^19^ show intrinsic fluorescence. No significant difference in the fluorescence peak position or intensity before and after aging could be observed for any sample concentration studied, as shown by the data in Figure S12. These results contrast with the pronounced shift in Trp fluorescence emission peak position and intensity noted for a semaglutide derivative with concentration 0.008 wt % in phosphate buffer solution.^19^ Our results indicate that the local environment of the Trp residue is not significantly altered upon micellization (or dimerization/trimerization at lower concentration). The critical micelle concentration (CMC) for semaglutide was obtained from Nile red fluorescence. The data in Figure S13 for a 45-day aged sample show a CMC (0.06 ± 0.005) wt %.

As a simple initial test of the influence of self-assembly on the bioactivity of semaglutide, we performed a transwell permeability assay, comparing the transepithelial electrical resistance (TEER) of fresh and aged samples using gut epithelial Caco-2 cells. The results in Figure 4 show that fresh semaglutide causes an initial decrease (after 30 min) of around 80% of the initial TEER with a slow but progressive recovery, similar to previous literature reports.^55^ The observed TEER decrease for aged semaglutide was less pronounced, around 90% of the initial TEER after 2–4 h, and also delayed in comparison to fresh semaglutide. These results suggest that unaggregated fresh semaglutide has more activity in permeating the gut epithelium than aged samples containing aggregates.

**Figure 4:**
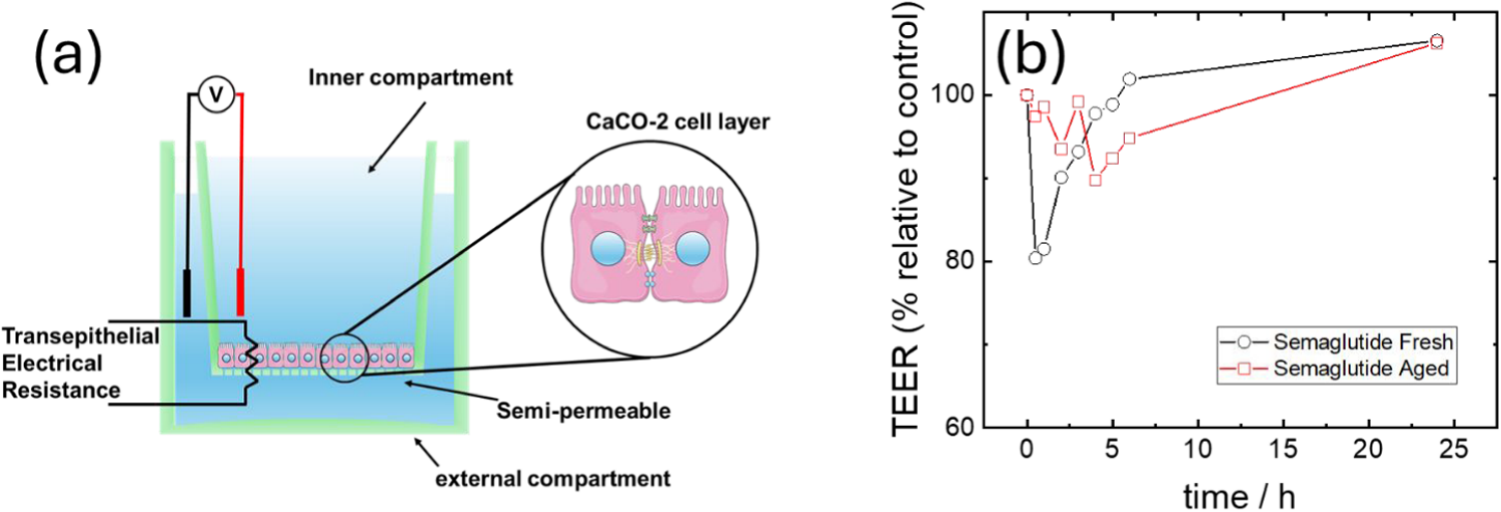
Transepithelial electrical resistance assays to probe Caco-2 cell membrane permeability. (a) Scheme of setup. (b) TEER percentage relative to DMEM control exposed to fresh and aged (40-day) semaglutide solutions (0.02 wt %).

## Conclusions

In summary, semaglutide in aqueous solution initially forms an irregular fractal-like structure, but after prolonged aging (40 days), micelles are observed at a sufficiently high concentration. There is an additional small population of needle-shaped fibrils. This structure does not evolve after a further period of aging, suggesting that the micelles are stable nanostructures. The micelles are stabilized by electrostatic interactions that can be described using a structure factor developed for charged colloid particles. The micelles have a 2 nm radius, as determined from cryo-TEM and SAXS, and the association number is estimated *at p* ≈ 30. Lower concentration samples (0.1 wt %) comprise dimers and trimers.

The observed changes in TEER of a Caco-2 cell membrane may result from self-assembly of semaglutide into micelles, which can decrease the amount of free semaglutide in the cellular microenvironment. This effect has been widely applied in controlled release systems for bioactive peptides, where the structures formed during the aggregation process (aggregate nanostructures, gel networks, etc.) are designed to slowly release the peptide, providing a delay in the desired effect. In contrast to the fibril formation observed for GLP-1 itself and other derivatives such as liraglutide, we have found that semaglutide forms micelle aqueous solution. The aggregation of other GLP-1 agonist lipopeptides on the market and in development, and their potential effect on bioactivities, merit further urgent investigation, as does the aggregation behavior of semaglutide in the presence of other salts or buffers, as in formulations used in therapeutic applications.

## Supplementary Material



## Notes

HPLC and ESI-MS data; additional cryo-TEM images; additional SAXS data; additional MD data; CD spectra; fluorescence spectra; and table of SAXS fitting parameters (PDF)
