# Supplementary material for: Semaglutide Aggregates into Oligomeric Micelles and Short Fibrils in Aqueous Solution
Source: Biomacromolecules. 2025 May 12;26(6):3786–94. doi: 10.1021/acs.biomac.5c00342 (PMC12152837; doi:10.1021/acs.biomac.5c00342)
Supplement: Supplementary file 1 [file bm5c00342_si_001.pdf]

Supporting Information

Semaglutide Aggregates into Oligomeric Micelles and Short Fibrils in Aqueous Solution

Ian W. Hamley,[1,*] Lucas R. de Mello, [1] Valeria Castelletto,[1] Thomas Zinn,[2] Nathan Cowieson,[2] Jani Seitsonen,[3] Thomas Bizien[4]

[1] *School of Chemistry, Food Biosciences and Pharmacy, University of Reading, Whiteknights, Reading, Berkshire, RG6 6AD, U.K.*

[2] *Diamond Light Source, Harwell Science and Innovation Campus, Didcot, Oxfordshire, OX11 0DE, I.K.*

[3] *Nanomicroscopy Center, Aalto University, Puumiehenkuja 2, FIN-02150 Espoo, Finland*

[4] *Synchrotron SOLEIL, L'Orme des Merisiers Départementale 128, 91190 Saint-Aubin, France*

* Author for correspondence: I.W.Hamley@reading.ac.uk

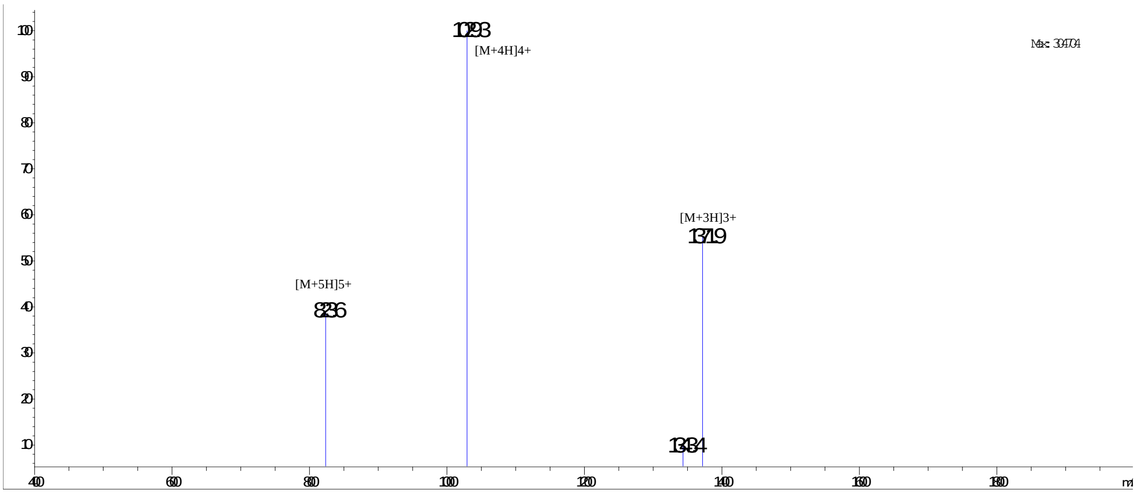

Sample Description		Instrument	Agilent-6125B		
Analyzed date:	2024-09-24	Probe:	ESI	Probe Bias:	＋4.5kv
Analyst:	YU	Nebulizer Gas Flow:	1.5L/min	Detector:	1.5kv
Sample:	Semaglutide	CDL:	-20.0v	T. Flow:	0.2ml/min
M.W.:	4113.64	CDL Temp.:	250 ^oC	B. Conc.:	50%H2O/50%ACN
Lot. No.:	P240629-20-YW1026338	Block Temp.:	200 ^oC		

SI Fig.S1. ESI-MS data for semaglutide.

Product Name: Semaglutide
Instrument No: 0200023
Lot No : P240629-20-YW1026338
Column :4.6*250mm,GS-120-5-C18-BIO
Solvent A :0.1%Trifluoroacetic in 100% Acetonitrile
Solvent B :0.1%Trifluoroacetic in 100% Water

Gradient :	A	B
0.00min	45%	55%
25min	70%	30%
25.1min	100%	0%
30min	Stop	

Flow rate :1.0ml/min
Wavelength :220nm
Volume :10ul

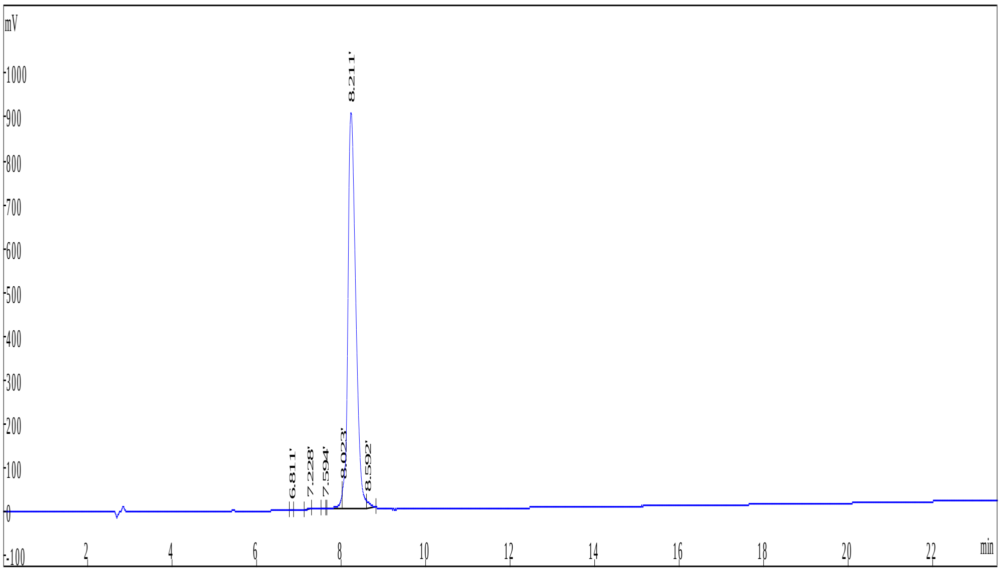

Rank	Time	Quantity	Area	Height
1	6.811	0.0090	998	320
2	7.228	0.0331	3651	738
3	7.594	0.0119	1310	408
4	8.023	1.1342	125070	41041
5	8.211	98.1404	10822466	898578
6	8.592	0.6714	74043	13878
Total		100	11027538	954963

SI Fig.S2. HLPC chromatogram for semaglutide.

(a) Fresh Semaglutide

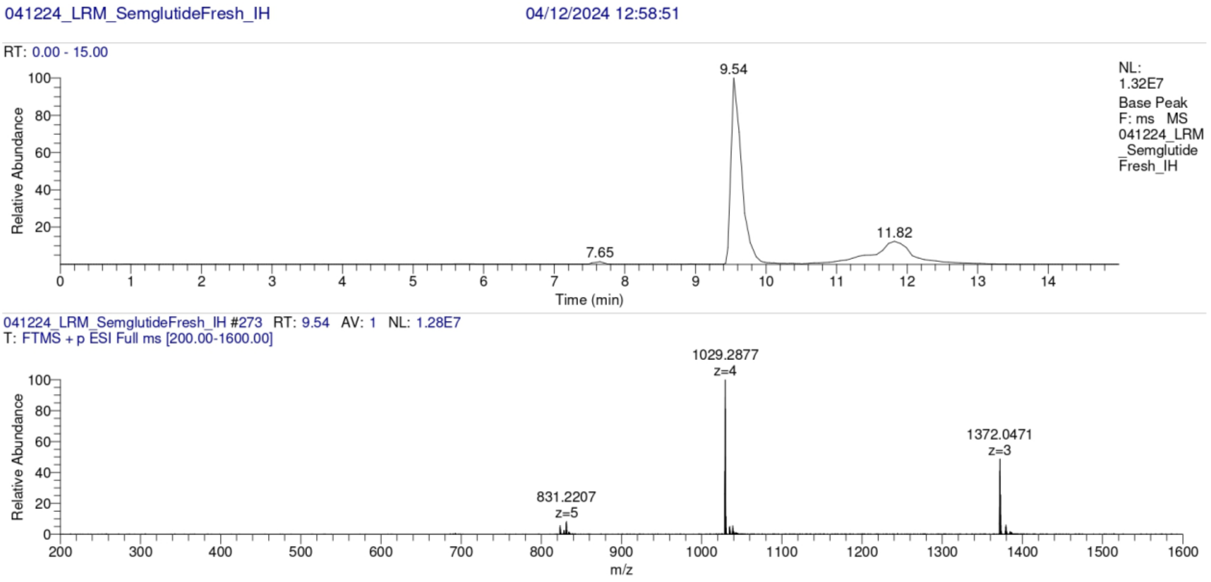

(b) 40 day semaglutide

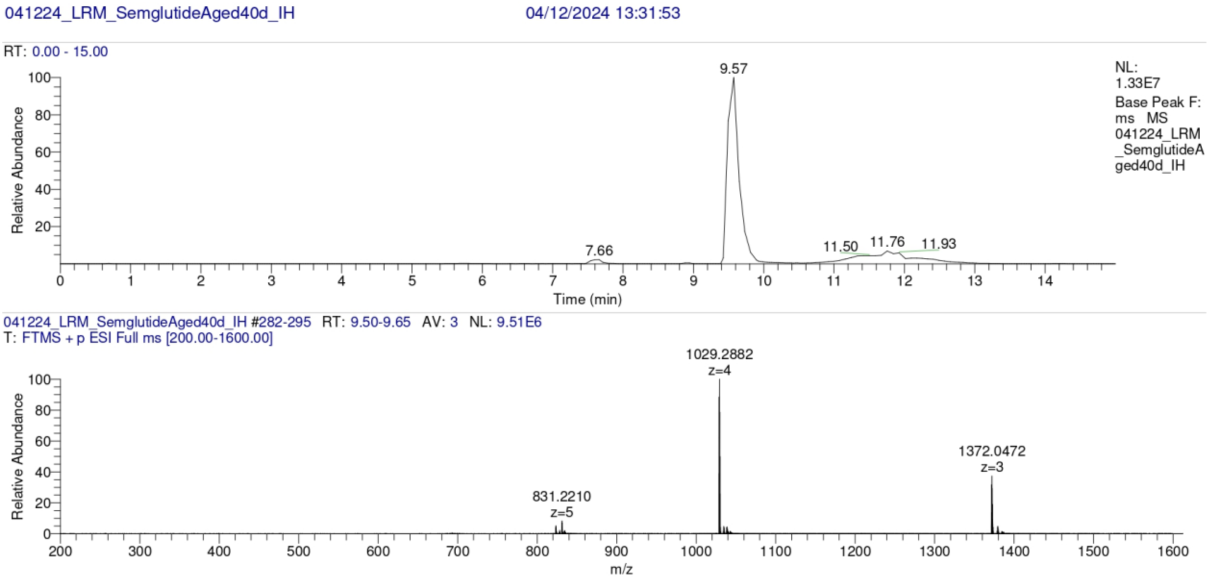

SI Fig.S3 ESI-MS data for semaglutide (measured in our labs) for (a) freshly prepared and (b) 40-day aged sample.

(a)

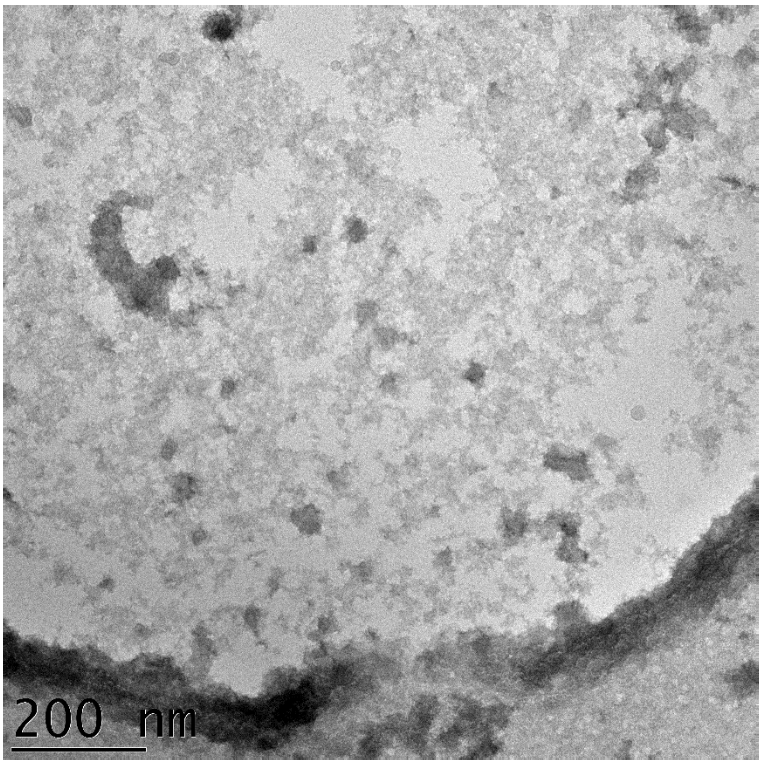

(b)

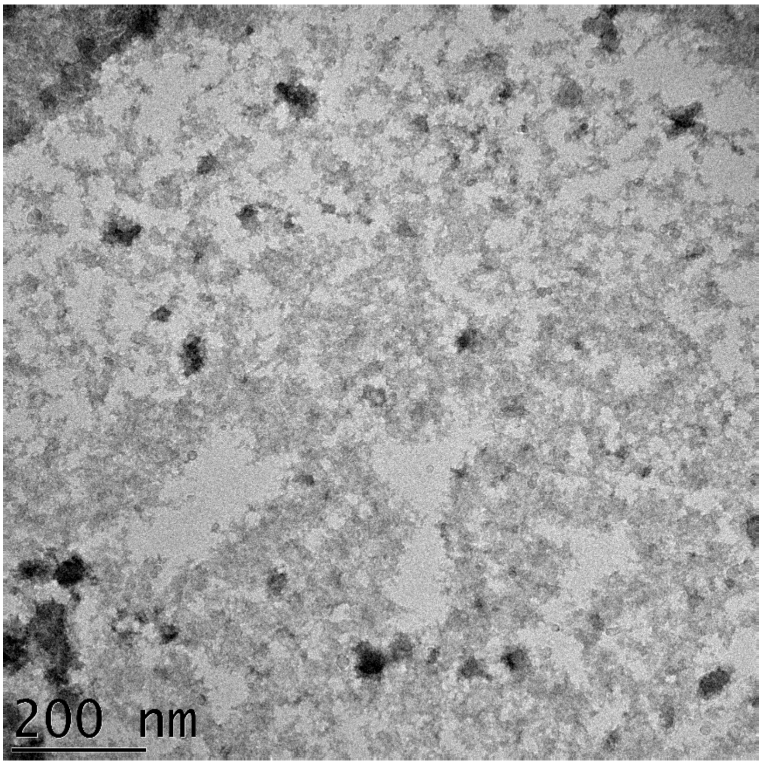

(c)

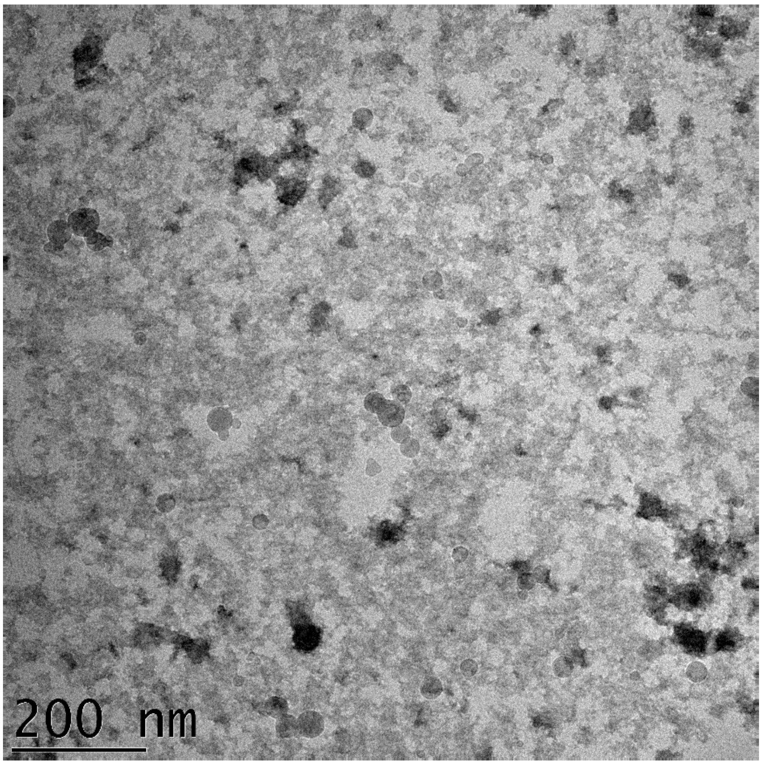

SI Fig.S4. (a-c) Additional cryo-TEM images (1 wt% semaglutide freshly prepared).

(a)

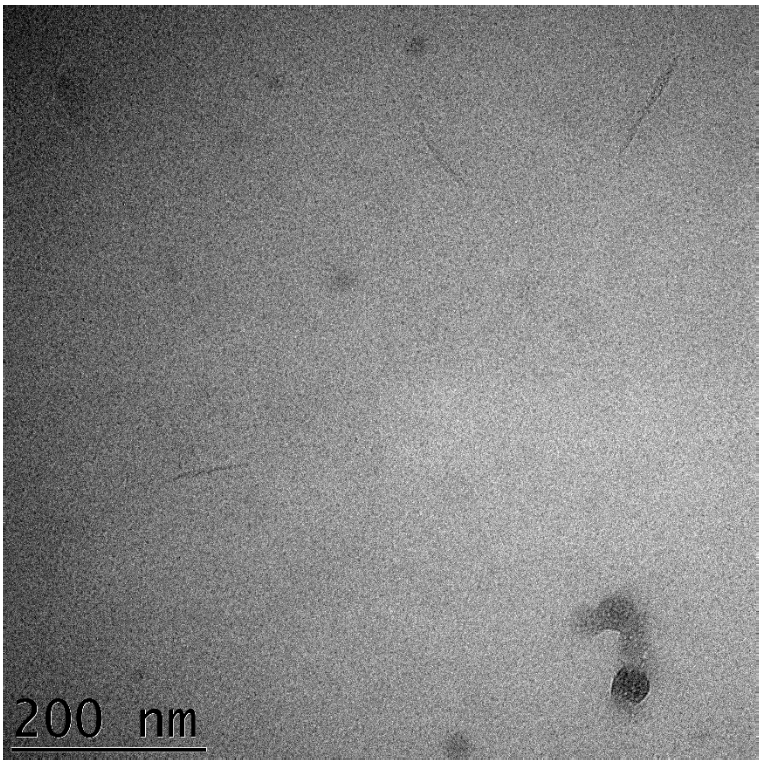

(b)

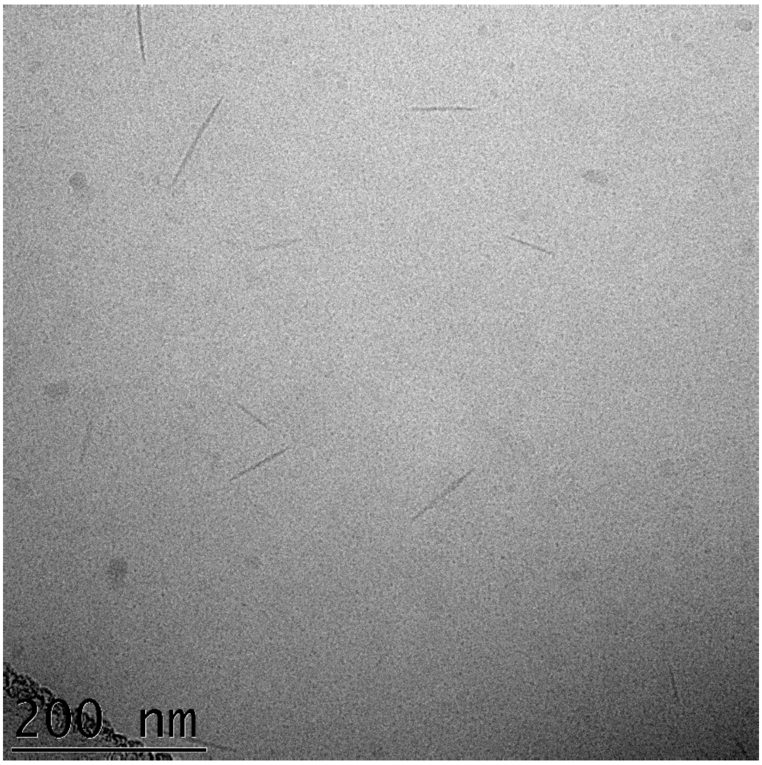

(c)

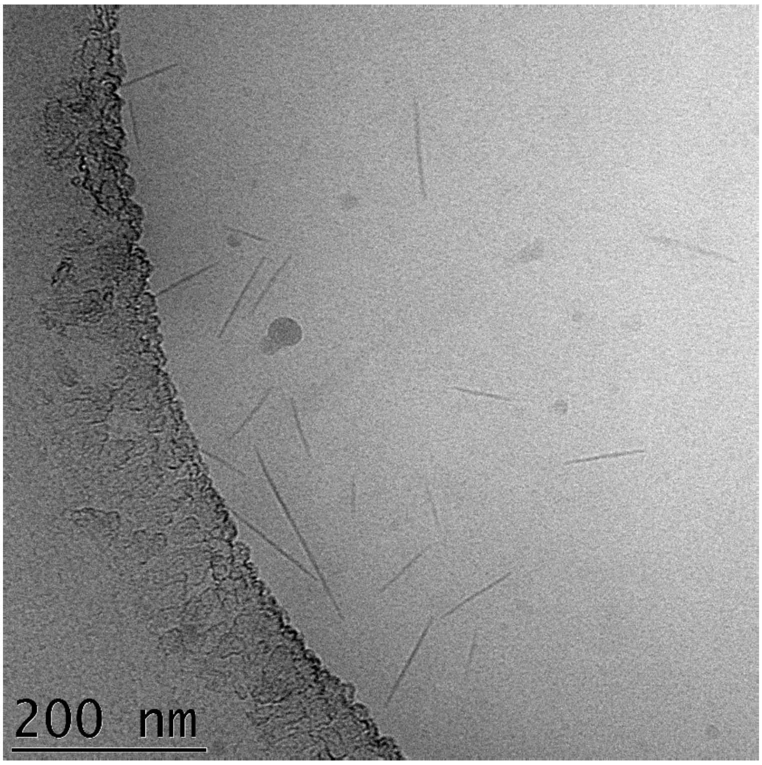

SI Fig.S5. (a-c) Additional cryo-TEM images (1 wt% semaglutide after 40 days).

(a)

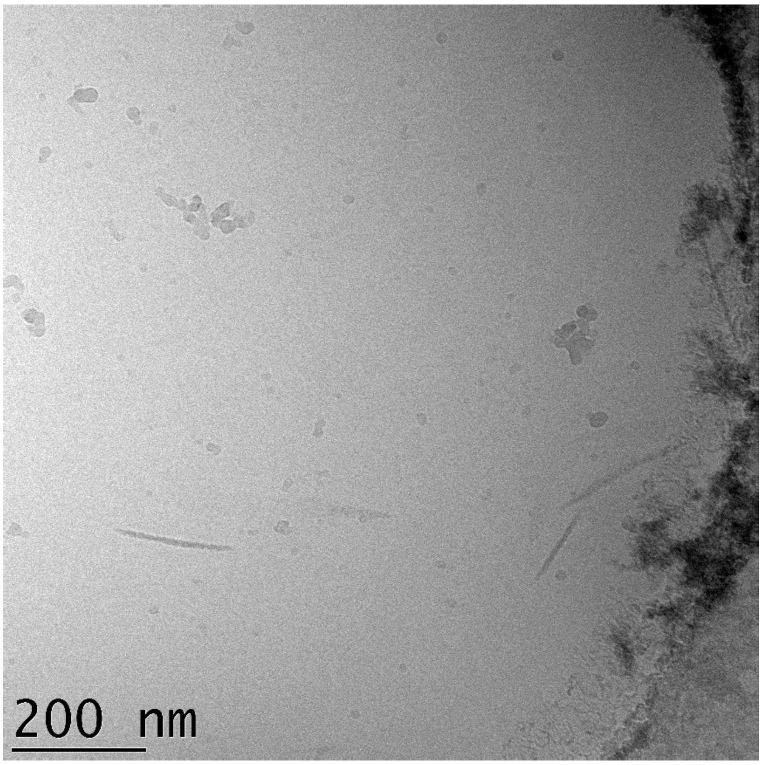

(b)

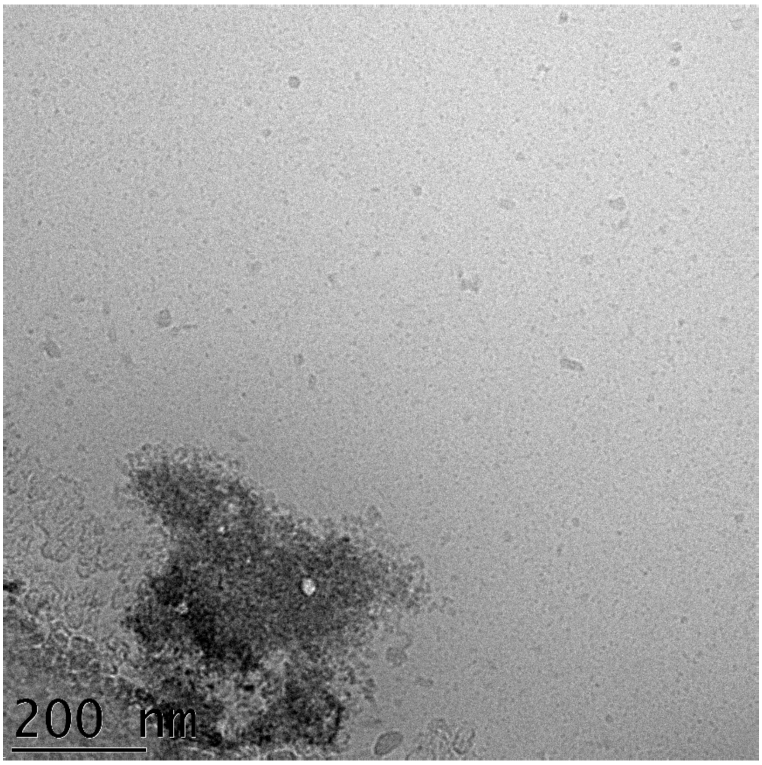

(c)

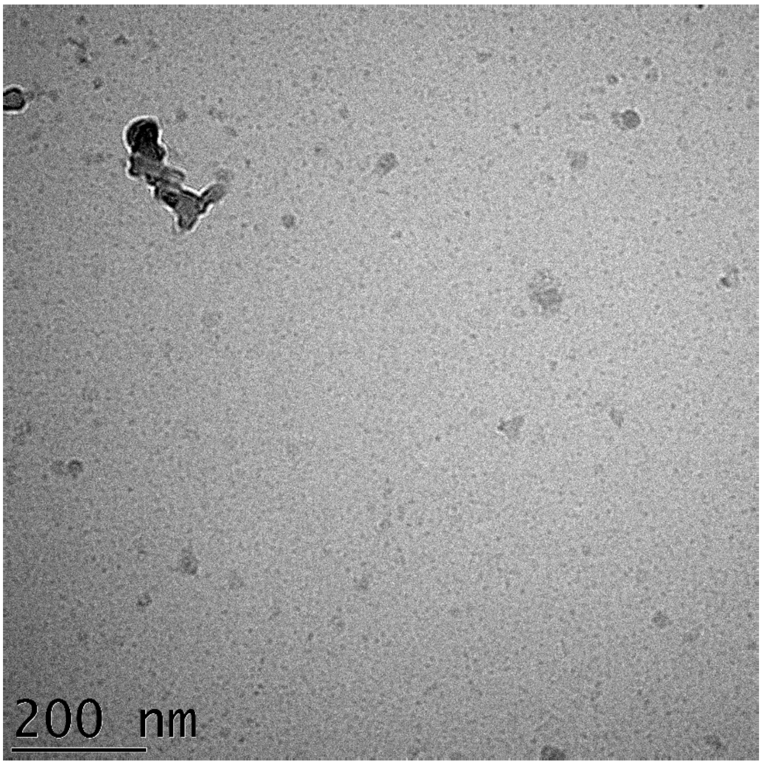

SI Fig.S6. (a-c) Additional cryo-TEM images (1 wt% semaglutide after 79 days).

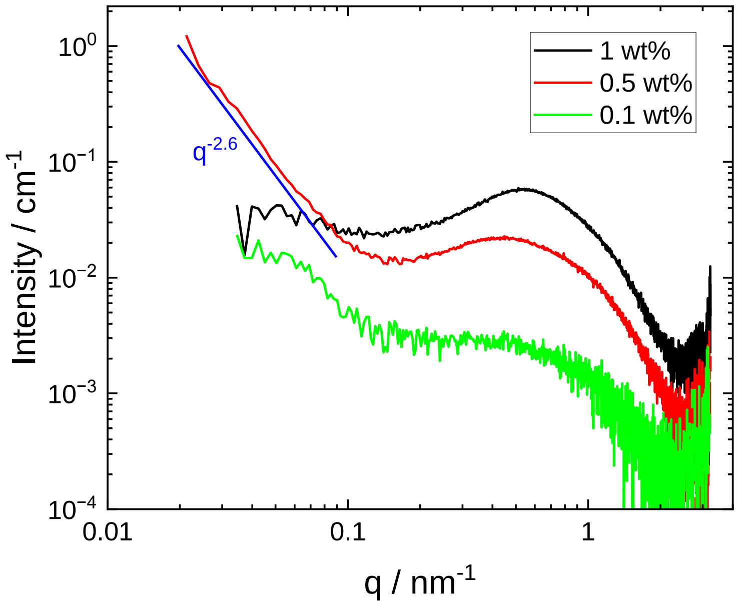

SI Fig.S7. SAXS data for fresh samples (5 days old), extending to lower *q* (measured on synchrotron beamline SWING).

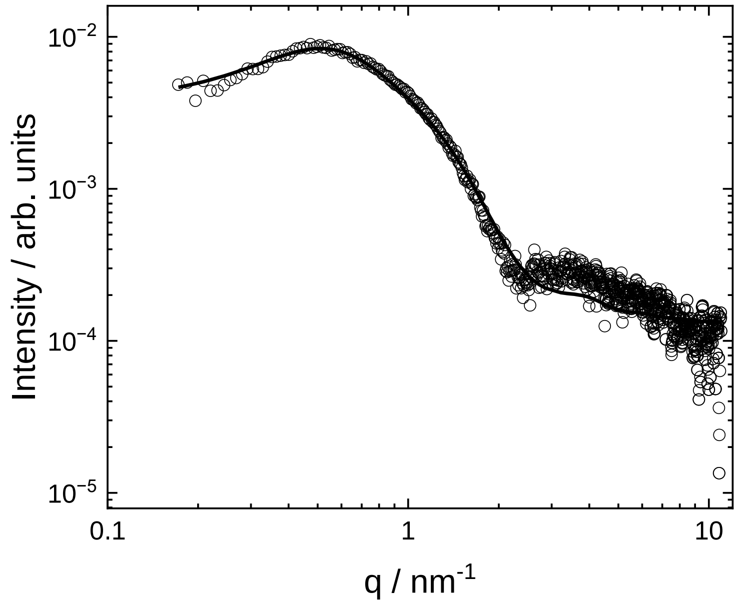

SI Fig.S8. SAXS data (open symbols) and fit described in text(parameters in SI Table S1) for aged 1 wt% sample sample, extending to higher *q* (measured on lab instrument, not in absolute units).

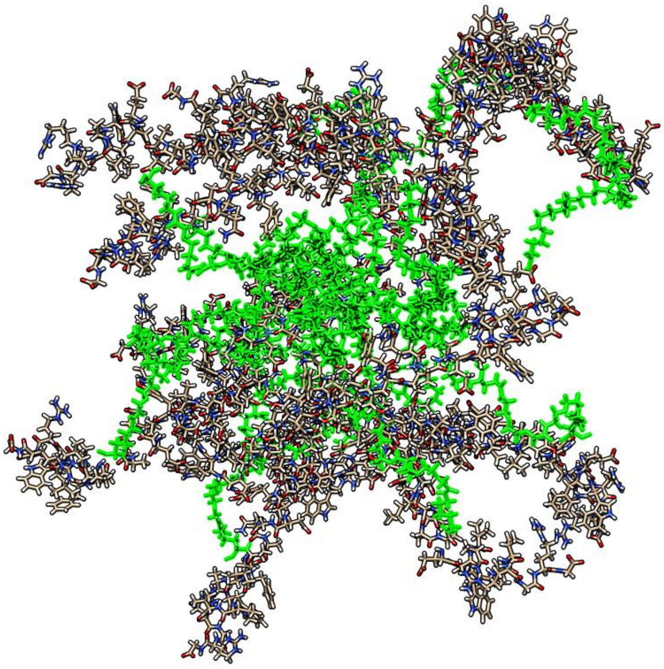

SI Fig.S9. Image of micelle from final frame of MD simulation of semaglutide with p = 15. The green chains are highlighted lipidated side chains on Lys20.

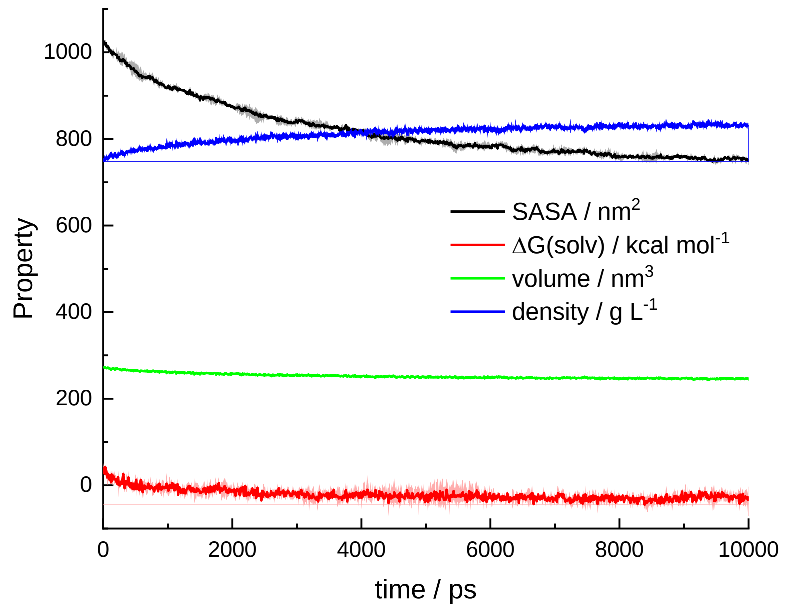

SI Fig.S10. Solvent-accessible surface area (SASA), Gibbs energy of solvation, volume and density computed from final 10 frames of MD simulations (3 repeats, maximal-minimal differences shown in light shades around solid lines) for semaglutide micelle with p = 30.

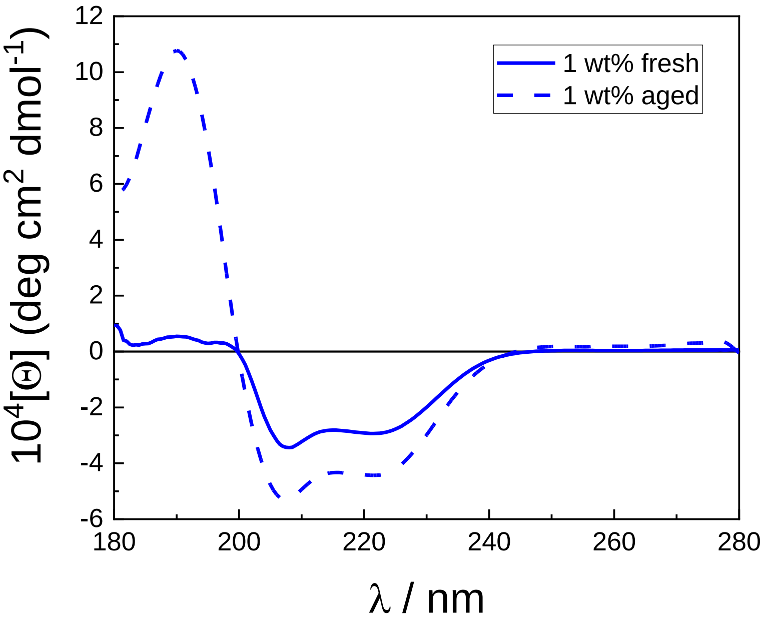

SI Fig.S11. Circular dichroism spectra for samples freshly prepared (day 0) and after 40 days aging.

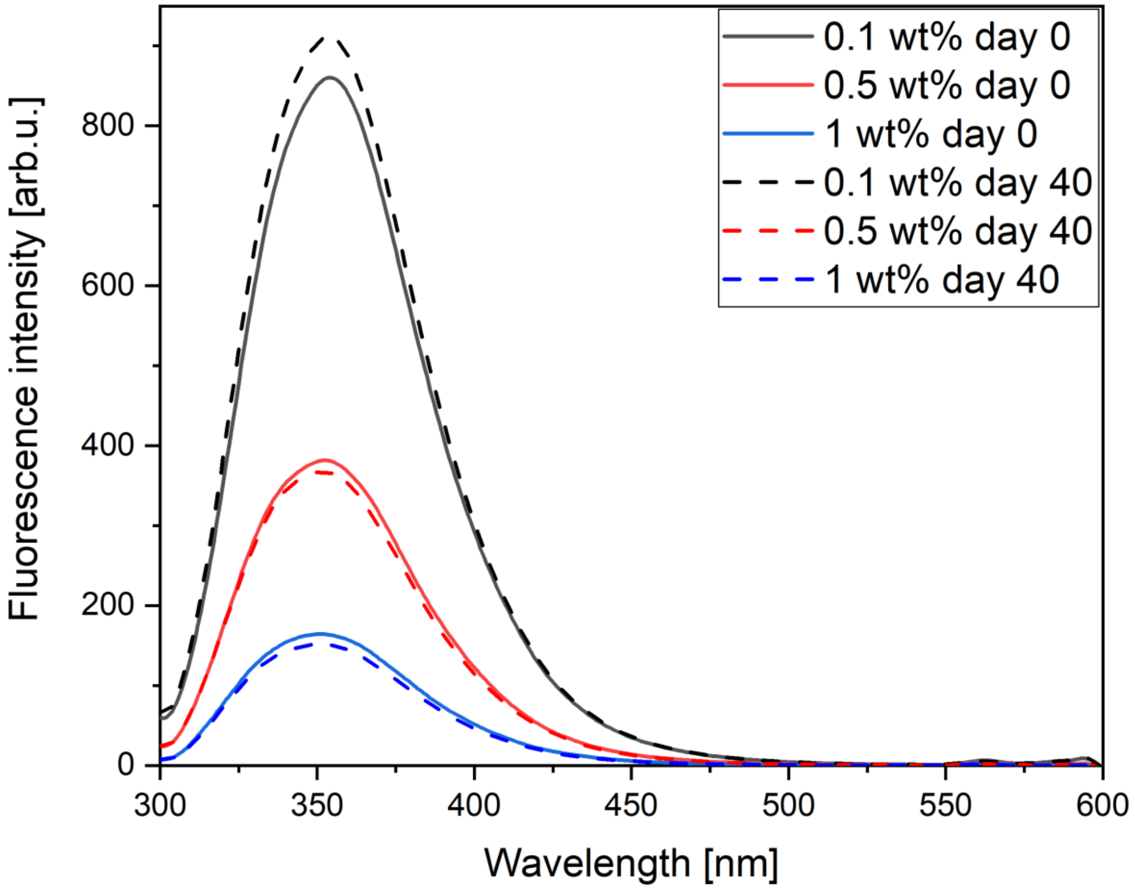

SI Fig.S12. Comparison of semaglutide intrinsic Trp fluorescence for fresh and aged samples.

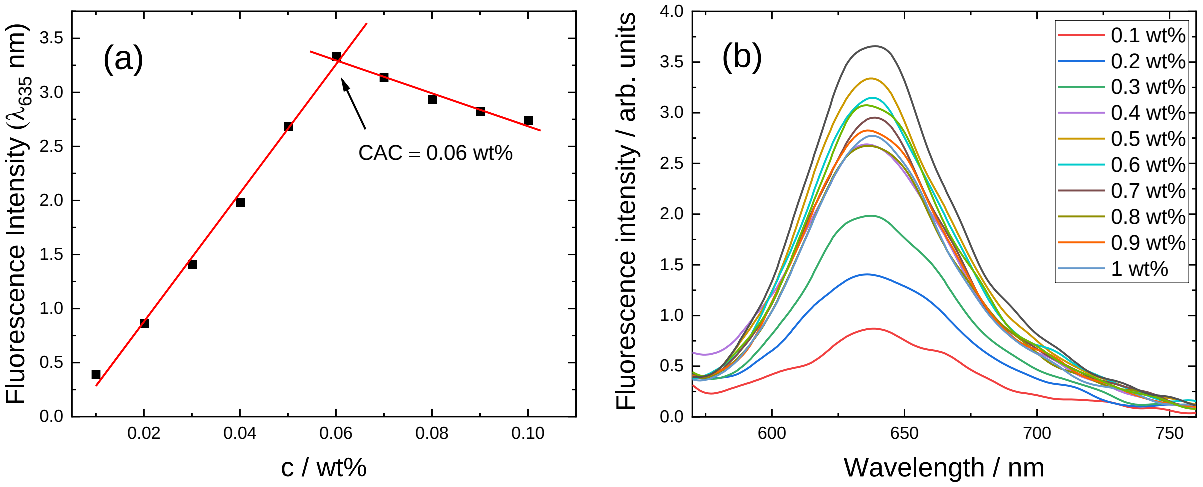

SI Fig.S13. Nile red fluorescence assay of critical micelle concentration (CMC) for a 45-day aged sample. (a) Plot of peak fluorescence intensity (λ = 635 nm) versus concentration, (b) Original fluorescence spectra.

Table S1. Fit parameters to the SAXS data in Fig.2b using a core-shell sphere form form factor with the Hayter-Penfold RMSA structure factor.[1] The monodisperse approximation to factor the form and structure factors of isotropic solutions of centrosymmetric particles is assumed. Fitting performed using SASfit.[2-3]

	**0.1 wt%** (Diamond synch.)	**0.5 wt%** (Diamond synch.)	**1 wt%** (Diamond synch.)	**1 wt%** (Lab SAXS)[b]
$R_o \pm \sigma$ / nm [a]	-	2.31±0.59	2.31±0.59	2.31±0.59
R_i / nm [a]	-	1.50	1.50	1.50
μ [a]	-	5.00	5.00	5.00
η / cm^{-1}	-	1.07×10^{-6}	1.86×10^{-6}	8.3×10^{-7}
R_{HS} / nm	-	2.23	2.54	2.54
ϕ	-	0.010	0.021	0.021
z_{eff}	-	9.81	10.8	9.00
I / M [a]	-	0.0073	0.0073	0.0073
BG	8.35×10^{-5}	7.65×10^{-4}	1.24×10^{-4}	1.24×10^{-4}
R_g / nm	1.57			0.8
I_c / cm^{-1}	2.93×10^{-3}			3×10^{-4}

Key: Core-Shell Sphere Form factor: R_o: outer radius (σ_c Gaussian polydispersity in R_0), R_i: inner radius, μ: ratio of scattering contrast of inner core/outer core, η: scattering contrast of core. **Structure factor**: R_{HS}, hard sphere radius, ϕ: effective volume fraction z_{eff}: effective charge modulus, I: ionic strength (temperature fixed at T = 293 K). Gaussian coils: R_g: radius of gyration, I_c: intensity. **Background**: BG (constant).

[a] Fixed for the fits at the two concentrations

[b] Same parameters as for synchrotron fits apart from scattering contrast (which scales data, allowing for non-absolute units) and z_{eff} (differs due to the broader instrument resolution function for the lab instrument which influences the structure factor peak width) and with additional Gaussian coil term to account for high q scattering.

References

(1) Hayter, J. B.; Penfold, J., An analytical structure factor for macroion solutions. *Molec. Phy.* **1981,** *42*, 109-118.

(2) Bressler, I.; Kohlbrecher, J.; Thünemann, A. F., SASfit: a tool for small-angle scattering data analysis using a library of analytical expressions. *J. Appl. Cryst.* **2015,** *48*, 1587-1598.

(3) Kohlbrecher, J.; Bressler, I., Updates in SASfit for fitting analytical expressions and numerical models to small-angle scattering patterns. *J. Appl. Cryst.* **2022,** *55*, 1677-1688.
